# Analysis of trunk muscles activity during horseback riding machine exercise in children with spastic cerebral palsy

**DOI:** 10.1097/MD.0000000000031915

**Published:** 2022-12-30

**Authors:** Kyeongbong Lee, JungHee Jung, HyeonHui Shin, KyungJune Lee, HyoSun Lee, GyuChang Lee

**Affiliations:** a Department of Physical Therapy, Kangwon National University, Samcheok, Republic of Korea; b Department of Physical Therapy, Graduate School of Kyungnam University, Changwon, Republic of Korea; c Department of Occupational Therapy, Dongseo University, Busan, Republic of Korea; d Department of Broadcasting and Communication Policy, Seoul National University of Science and Technology, Seoul, Republic of Korea; e Department of Physical Therapy, Kyungnam University, Changwon, Republic of Korea.

**Keywords:** cerebral palsy, horseback riding machine, trunk muscles

## Abstract

Appropriate trunk muscle activity is needed to perform functional activities in cerebral palsy, this study analyzed the activity of trunk muscles during horseback riding machine exercise in children with spastic cerebral palsy. 10 children with spastic cerebral palsy were participated, the activity of the trunk muscles, including both sides of the rectus abdominis, external oblique, latissimus dorsi, and erector spinae in sitting posture and during horseback riding machine exercise were evaluated using a surface electromyography. The activity of bilateral rectus abdominis, external oblique, latissimus dorsi, and erector spinae increased during horseback riding machine exercise than quiet sitting posture. Moreover, there were significant differences in activities of the trunk muscles between the sitting posture and horseback riding machine exercise, with the exception of the left external oblique and the left latissimus dorsi. Horseback riding machine exercise provides more opportunities to use the trunk muscles for children with spastic cerebral palsy than general sitting posture. In future, it will be of use to investigate the effect of horseback riding machine exercise in patients with cerebral palsy.

## 1. Introduction

Cerebral palsy is a syndrome that manifests as non-progressive abnormalities due to lesions or damage occurring in developing brains before, during, or after birth.^[1]^ As medical technology advances, the live birth rates of premature infants had increased, and the incidence of cerebral palsy has also gradually increased due to brain prematurity or damage. The main symptoms of cerebral palsy are lesions in motor functions, posture, and movement, and may accompany issues in cognition, communication, and learning, depending on the area and degree of brain damage.^[2]^ One of the most important problems in children with cerebral palsy is the defect in postural balance. Maintain the sitting balance is necessary to perform the independent activities of daily living, the lowered postural balance is a major challenge for children with cerebral palsy.^[3]^

As the muscular strength and proprioception of the trunk decrease, the ability of the trunk to control balance decreases.^[3,4]^ When the body posture changes or trunk stability is required, if the muscular strength of the trunk is reduced, it makes difficult to maintain the adequate posture due to postural sway being increased.^[5]^ As the trunk control are maintained by antigravity activation of the trunk muscles, proper activity of the trunk muscles is essential for functional use of upper and lower extremities.^[6,7]^ Proper activity of the trunk muscles is related to the maintenance of trunk stability and the level of functional use of the lower extremities.^[8]^ To maintain a sitting posture, children with cerebral palsy perform stereotypical activation of extensor muscles, abnormal muscle recruitment, and excessive activation of antagonists.^[9–11]^ These components reduce the quality of functional activities of upper and lower extremities such as reaching movement and maintenance of sitting posture which can be performed by appropriate postural control. Thus, the children with cerebral palsy need to perform exercises those can enhance the trunk stability, but it is difficult to properly achieve smooth and functional movements. Thus, it is crucial to maintain adequate strength and endurance of trunk muscle in order to keep the sitting postures and functional movements in activities of daily living.

Among the various interventions to enhance trunk stability, there are hippotherapy for children with cerebral palsy has been reported.^[12–14]^ Although there are many advantages of hippotherapy, the use of living horses has several limitations including high costs, lack of access to facilities, deficiency of infrastructures, and the requirement for professional coaches.^[15]^ In order to make up for such limitations while retaining the advantages of horseback riding, some attempts have been made to incorporate a real horse-like horseback riding machine into clinical practice.^[16]^ Various horseback riding machines were developed to allow people to take horseback riding exercise indoors, most of the studies on horseback riding machine exercise have investigated the enhancement of balance and walking ability in children with intellectual disability.^[17–19]^ However, there have been no studies on the activity of trunk muscles in children with cerebral palsy during horseback riding machine exercise.

Therefore, the aim of this study is to evaluate the activity of trunk muscles those can affect trunk stability in children with cerebral palsy to provide a reference for the trunk balance exercise program.

## 2. Methods

### 2.1. Participants

In this study, the participants were children diagnosed with cerebral palsy who were recruited through a bulletin board in the S Development Institution located in South Korea. The inclusion criteria were as follows: children diagnosed with spastic cerebral palsy; children whose grades of gross motor functioning classification system were I, II, and III; children aged 6 to 12 years; and children who could understand and follow instructions provided by the researcher. A total of 10 children were recruited, and their general information was as follows: boys (n = 7) and girls (n = 3); average age, 8.5 (2.55) years; average height and weight, 125.2 (13.2) cm and 29.4 (12.62) kg, respectively.

### 2.2. Experimental procedures

The present study is a cross-sectional study to determine the changes in trunk muscles activity that affects trunk stability during horseback riding machine exercise. The participants’ general information, including sex, age, height, and weight, was collected through brief interviews. The trunk muscle activity of the participants was measured in the sitting posture and during horseback riding machine exercise. First, the maximum voluntary isometric contraction (MVIC) was measured, and the electromyography signals of the trunk muscle activity were normalized to peak activity in the MVIC trial and expressed as a percentage. The participants took a break for 10 minutes between each measurement to prevent bias due to fatigue which could occur during exercise or measurement. Then, the participants were asked to sit in a chair with feet touching the ground and eyes fixed on a point 1 m ahead of them. The activity of the trunk muscles was measured three times every 10 seconds; after a 10-minute break, they were asked to sit on the horseback riding machine.

**Figure 1.**
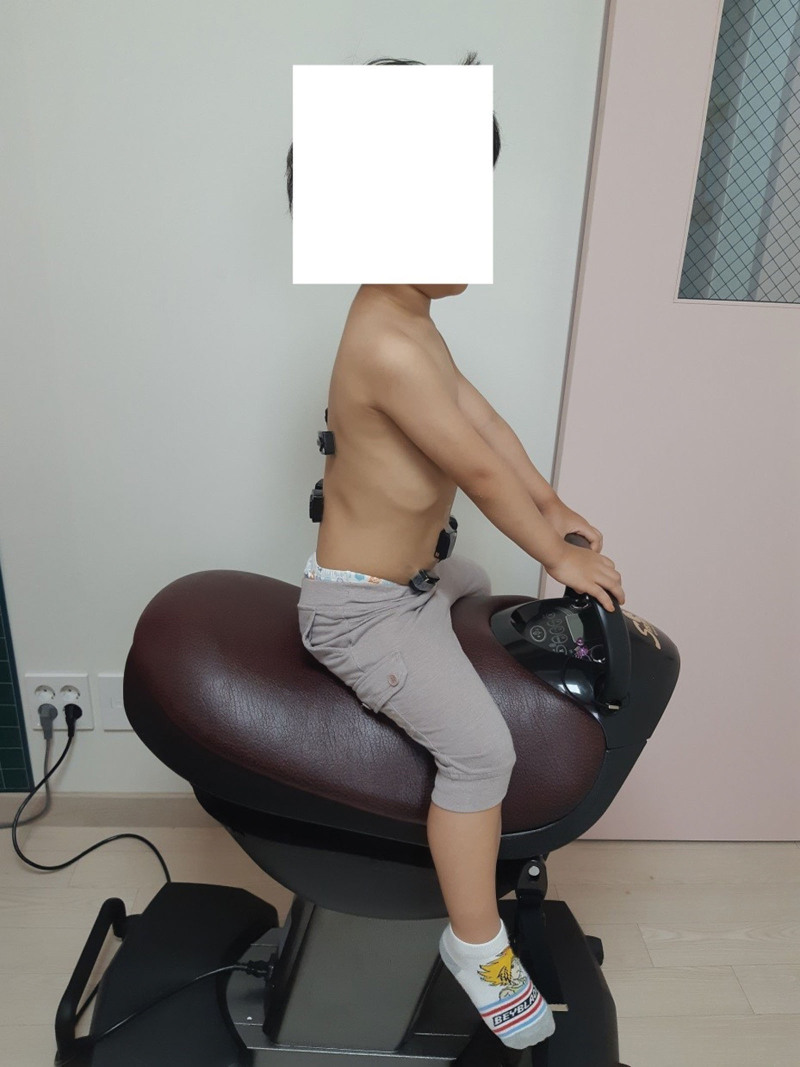
Horseback riding machine exercise.

The horseback riding machine (Luxury, S-RIDER, Sejong, Republic of Korea) used in this study is a luxury model for indoor use, produced by the S-RIDER company (Fig. 1). The equipment measures 74 cm high, 70 cm long, and 45 cm wide, and contains five autonomous exercise programs so that users can conveniently select from a variety of exercise programs.

In order to reduce the fear of horseback riding machine exercise, the research method and procedure were explained before the measurement. With the supervision of the research assistants and caregivers, the height of the support was adjusted to the level on which they could easily climb to the saddle of the horseback riding machine. The participants were asked to climb on the horseback riding machine, stably sit on the saddle while spreading their legs in a standing posture, and spread their legs after putting their hips on it and turning their bodies. Among the exercise programs of the horseback riding machine, abdominal muscle strengthening was selected and performed at a level 2 strength. Following the initiation of the horseback riding exercise, muscle activity was measured three times every 10 seconds. To prevent accidents that may occur during the experimental process, mattress was put around the horseback riding machine, and research assistants carefully observed the subjects at a very close distance during the experiment.

### 2.3. Electromyography analysis

A surface electromyogram (Trigno EMG Sensor, Delsys Inc., Boston, MA) was used to measure the trunk muscles activity in the sitting posture and during horseback riding machine exercise. To gain the electromyography signal for trunk muscle activity, four pairs of surface electrodes were attached to the right and left sides of the trunk. The areas were wiped before the electrodes were attached to them in order to minimize resistance generated from the skin. Each electrode was attached according to the SENIAM (surface electromyography for the non-invasive assessment of muscle) guideline. The activities of the rectus abdominis, external oblique, latissimus dorsi muscle, and erector spinae were measured by attaching electrodes to them after accurately marking the positions of the centers of each muscle curve with a water-resistant pen.

The analog signals of each muscle were measured using electromyography electrodes and the signals were wirelessly transmitted to the Trigno base station. The analog signals were subsequently converted to digital signals, and then collected and processed with Delsys EMG Works Acquisition on personal computers. The sample rate of electromyography signals was set as 2000 Hz, and a frequency bandwidth of 20 to 40 Hz was used to eliminate the noise of electromyography signals. The collected signals were calculated as the root mean square. The electromyography signals for a total of 10 seconds were collected, and only the signals for the middle 3 seconds except for the first 3 seconds and the last 3 seconds were used in this study.

### 2.4. Statistical analysis

The data were statistically analyzed by using SPSS version 18.0 (IBM Corporation, Armonk, NY). The participants’ general information was analyzed with descriptive statistics. The activity of the trunk muscles in the sitting posture and during horseback riding machine exercise was compared using a paired *t* test. The significant differences were at *P* < .05.

### 2.5. Ethical approval

This study was approved by Institutional Review Board of Kyungnam University (Approval number: 1040460-A-2019-051), all methods were carried out in accordance with relevant guidelines and regulations. The physical therapists of the S Development Institution conducted this research after obtaining permission from the participants’ parents. All participants were informed of the purpose of the present study before complete the informed consents. All procedure of this study were carried out based on the ethical principles in the Helsinki Declaration.

## 3. Results

The results are shown in Table 1. The muscle activity of the rectus abdominis, right erector spinae, right latissimus dorsi, and erector spinae was significantly developed during horseback riding machine exercise compared to that in the sitting posture (*P* < .05). The activity of the rectus abdominis was increased from 23.75% to 51.16% (right) and from 36.61% to 62.97% (left) during horseback riding machine exercise compared to that in the sitting posture; that of the external oblique was increased from 32.60% to 59.19% (right), and from 35.90% to 52.29% (left); that of the latissimus dorsi muscle was increased from 29.75% to 51.26% (right), and from 31.02% to 52.23% (left); and that of the erector spinae from 31.23% to 54.76% (right), and from 30.65% to 50.57% (left).

**Table 1:** Analyzing and comparison of trunk muscle activities between sitting posture and horseback riding machine exercise.

Muscles		Sitting posture (%)	Horseback riding machine exercise (%)	*P*
Rectus abdominis	Rt.	23.75 (11.78)	51.16 (18.13)	.01*
	Lt.	36.61 (21.17)	62.97 (18.64)	.02*
External oblique	Rt.	32.60 (13.74)	59.19 (21.2)	.01*
	Lt.	35.90 (28.38)	52.29 (28.59)	.08
Latissimus dorsi	Rt.	29.75 (17.23)	51.26 (17.34)	.00*
	Lt.	31.02 (18.6)	52.23 (20.82)	.16
Erector spinae	Rt.	31.23 (15.73)	54.76 (15.8)	.00*
	Lt.	30.65 (17.34)	50.57 (22.91)	.03*

Values (%MVIC) are presented as mean (standard deviation).

MVIC = maximum voluntary isometric contraction.

* Significant differences on trunk muscle activities between sitting posture and horseback riding machine exercise were presented (*P* < .05).

## 4. Discussion

Trunk muscles participate in sitting balance and proper activity of the trunk muscles is required to perform voluntary movements.^[20,21]^ The trunk muscles activate automatically to body movements or sway,^[22]^ and are involved in postural control during the controlled movement of the limbs and trunk.^[23]^ The trunk is the central part of the midsagittal plane of the body that enables to maintain balance, it can make the proper sitting, standing, change of positions, and automatic control of posture when walking.^[24]^ Thus, it is necessary to ensure that adequate level of the trunk muscles activity for performing the functional activities.

There are various methods for measuring changes in muscle activity that affect trunk balance, several preliminary studies have measured the activity of trunk muscles during horseback riding machine exercise. The increased activity of the rectus abdominis and the erector spinae were reported by horseback riding machine exercise in a child with scoliosis for 5 weeks.^[25]^ It was suggested that the trunk muscle activity is increased during horseback riding machine exercise because the spine and pelvis stabilize the trunk in response to the movement of the riding machine.^[26]^ The action of various trunk and pelvis muscles play an important role in controlling the posture of the trunk, by enhancing the stability of the spine and pelvis during functional movement.^[27]^

On the horseback riding machine, the children were postulated to reduce the center of gravity displacement and keep the sitting posture safely on a moving surface in order to maintain the center of gravity within the support during riding. Multiple sensory inputs and centrifugal motion outputs of the central nervous system are continuously stimulated throughout the horseback riding machine exercise to ensure sitting posture and weight shift occurs in response to the rhythmic movement of horseback riding, it can improve the trunk stability, equilibrium response, and vertical alignment correction. This improves postural control by activating trunk muscles that are responsible for maintaining proper position against gravity.^[27–29]^ Repetition of this adjustment causes muscle strengthening for the trunk muscles, which leads to improved trunk balance and postural control against gravity.^[30,31]^ Horseback riding machine exercise is used to generate autonomic movement for the postural control thus the primary motor area opposite the basal nucleus, and the auxiliary motor area activate the postural-controlling muscles and provide the signal for the contraction of the acting muscle by shifting the center of gravity and causing postural sway throughout the body.

In the present study, the activities of the rectus abdominis, external oblique, latissimus dorsi muscle, and the erector spinae are significantly higher in horseback riding machine exercise than quiet sitting due to the length-tension and force-velocity relationship. It is thought that the exercise increases the stability of the trunk by inducing isometric contraction of the trunk muscles, and the length of the abdominal muscles around the spine is restored effectively. Due to the reclining motion of the horseback riding machine exercise, the muscles of the trunk are relatively elongated and the movement velocity of the spine and pelvis which vary depending on the saddle movement, thus the elongated trunk muscles are automatically activated to maintain the trunk posture.^[32]^ The sarcomere is added to elongated muscles in parallel according to the length-tension curve, the elongated muscles exert stronger power than resting state of the muscles.^[33]^ In other words, this phenomenon means that the more actin-myosin cross bridges are formed, the stronger force can be generated by the muscles. Thus, it is reasonable that stronger isometric contractions occur during horseback riding machine exercise, resulting in increased muscle activity. The results of the present study also showed that the trunk muscles activity that of examined in this study, rectus abdominis, the right external oblique and latissimus dorsi muscle, and erector spinae was significantly increased compared to that in the sitting posture, it can be thought that horseback riding machine can effectively stimulate the trunk musculature and improve the trunk stability.

The horseback riding exercise is one of the whole-body trainings, and it is needed the motor control and muscular strength for maintaining posture during exercise. According to preliminary studies on horseback riding exercise, movement and coordination of the trunk are enhanced by horseback riding exercise. Thus, functional recovery, muscle strength and endurance are increased through horseback riding exercise which helps to maintain normal muscle tone.^[34]^ In a study evaluating sitting balance by applying a horseback riding simulator to 40 spastic cerebral palsy, they reported that postural control in sitting position demonstrated meaningful improvement and suggested that the horseback riding simulator had higher motor functionality and better acceptance of therapeutic intervention.^[35]^ Therefore, horseback riding exercise can be a useful treatment method to activate muscle activity, and it is considered that various studies are needed for improve the sitting balance and postural control of polio patients.

This study has several limitations. First, the number of participants was small, and the age range was limited to 6 to 12 years, the results cannot be generalized to the children with cerebral palsy. It is needed to study the effect of the horseback riding machine exercise with a larger number of children with cerebral palsy in different age groups in future studies. Second, since only immediate effects were measured and compared by a cross-over design, a long-term follow-up will be required to investigate the sustainability of the effects of the horseback riding machine exercise. Third, since this study only targeted children with spastic cerebral palsy, further researches should be continued on horseback riding machine exercise are needed to include the other types of cerebral palsy. Based on the results of this study, it is considered that a follow-up study on the various effects of horseback riding machine exercise is necessary in the future.

## 5. Conclusion

The activities of the rectus abdominis, external oblique, latissimus dorsi muscle, and erector spinae in children with spastic cerebral palsy during horseback riding machine exercise were significantly higher than those in the sitting posture. The findings show that horseback riding machine exercise can lead to positive changes in the trunk muscle activities in children with cerebral palsy. These results provide one of the basic data for evidence on the benefits of horseback riding machine exercise for children with cerebral palsy. Future studies need to investigate the effects of horseback riding machine exercise on children with cerebral palsy.

## Author contributions

KL, JJ, and GL were involved in the conception and/or design of the study.

JJ and HS were involved in the acquisition and analyzation of the data.

KL, JJ, and GL were drafted the manuscript.

**Conceptualization:** Kyeongbong Lee, JungHee Jung, GyuChang Lee.

**Data curation:** JungHee Jung.

**Formal analysis:** Kyeongbong Lee, HyeonHui Shin, KyungJune Lee, HyoSun Lee.

**Investigation:** JungHee Jung.

**Supervision:** HyeonHui Shin, KyungJune Lee, HyoSun Lee, GyuChang Lee.

**Validation:** GyuChang Lee.

**Writing – original draft:** JungHee Jung.

**Writing – review & editing:** Kyeongbong Lee, GyuChang Lee, KyungJune Lee, HyoSun Lee.

## Correction

GyuChang Lee’s affiliation has been corrected from affiliation d to affiliation e.
