# Analysis of trunk muscles activity during horseback riding machine exercise in children with spastic cerebral palsy: Erratum

**DOI:** 10.1097/MD.0000000000032762

**Published:** 2023-01-20

In the article, “Analysis of trunk muscles activity during horseback riding machine exercise in children with spastic cerebral palsy,”^[1]^ which published in Volume 101, Issue 52 of *Medicine*, KyungJune Lee and HyoSun Lee have been added to the author list.

“Department of Broadcasting and Communication Policy, Seoul National University of Science and Technology, Seoul, Republic of Korea” has been added as affiliation d, shifting “Department of Physical Therapy, Kyungnam University, Changwon, Republic of Korea” to affiliation e. The author list has been updated accordingly.
